# A survey of pre-weaning calf management in Norwegian dairy herds

**DOI:** 10.1186/s13028-021-00587-x

**Published:** 2021-05-06

**Authors:** Julie Føske Johnsen, Ingrid Hunter Holmøy, Ane Nødtvedt, Cecilie Marie Mejdell

**Affiliations:** 1 Department of Animal Health and Food Safety, Norwegian Veterinary Institute, Sentrum, P.O. Box 750, 0106 Oslo, Norway; 2 Department of Production Animal Clinical Sciences, Norwegian University of Life Sciences, Sentrum, P.O. Box 369, 0102 Oslo, Norway

**Keywords:** Housing, Milk, Questionnaire, Welfare

## Abstract

The knowledge on dairy calves’ needs with regards to milk feeding and social housing is expanding but to be able to make improvements, knowledge is needed on how calves are managed at present in Norway. The aim of this study was to describe selected milk-feeding practices and social housing procedures for young (pre-weaning) dairy calves in Norwegian herds. A short questionnaire was distributed to a random sample of 912 dairy producers. For each herd, we asked how much milk was fed to 3 week old dairy calves, number of daily milk feedings, usage of milk replacer, number of weeks calves were housed in single pens, and whether calves at 3 weeks had free access to drinking water. In total, 508 herd managers responded to the questionnaire (56% response rate). Descriptive statistics showed that median milk allowance for 3 week old calves was seven (IQR 6–8) L milk/d, ranging from 2 to 15 L/day. Consequently, 311 (61%) herds reportedly fed less than the current industry recommendation in Norway (8 L milk/d to young calves). Automatic milk feeders were used by 30 herds (6%). In herds feeding milk manually (n = 471), half of the herds (50%) fed milk twice daily. Median number of daily milk feedings was 3 (31%) while 13% fed four times/day or more frequent (6%). Of the 226 producers (46%) who reported to use milk replacer, this milk type was used from the calf age of (median) 2 weeks. Of all herds, 82 (16%) did not provide their 3 week old calves with free access to drinking water. In the surveyed herds, calves were housed in single pens for (median) 2 weeks (IQR 2–3), while legislation allows single housing until the age of 8 weeks. In conclusion, the milk allowances reported in this survey are low compared to industry recommendations which again are low compared to voluntary intakes of young dairy calves. Free access to drinking water is important to calf welfare but was not granted to all dairy calves. Most calves were reportedly group housed at an early age, which indicates an improved awareness with regards to the importance of social housing.

**Supplementary Information:**

The online version contains supplementary material available at 10.1186/s13028-021-00587-x.

## Findings

Best practice on feeding and housing artificially reared calves is changing along with expanding knowledge [1]. Feeding restricted amounts of milk (~10% of their body weight) to dairy calves housed in individual pens has been common practice in many countries since the 1950’s [2]. New research points to benefits in socially housing the calf [3] and increasing calf growth rates during the pre-weaning phase [4]. To be able to make improvements, knowledge is needed on how today’s calves are managed pre-weaning.

**Fig. 1:**
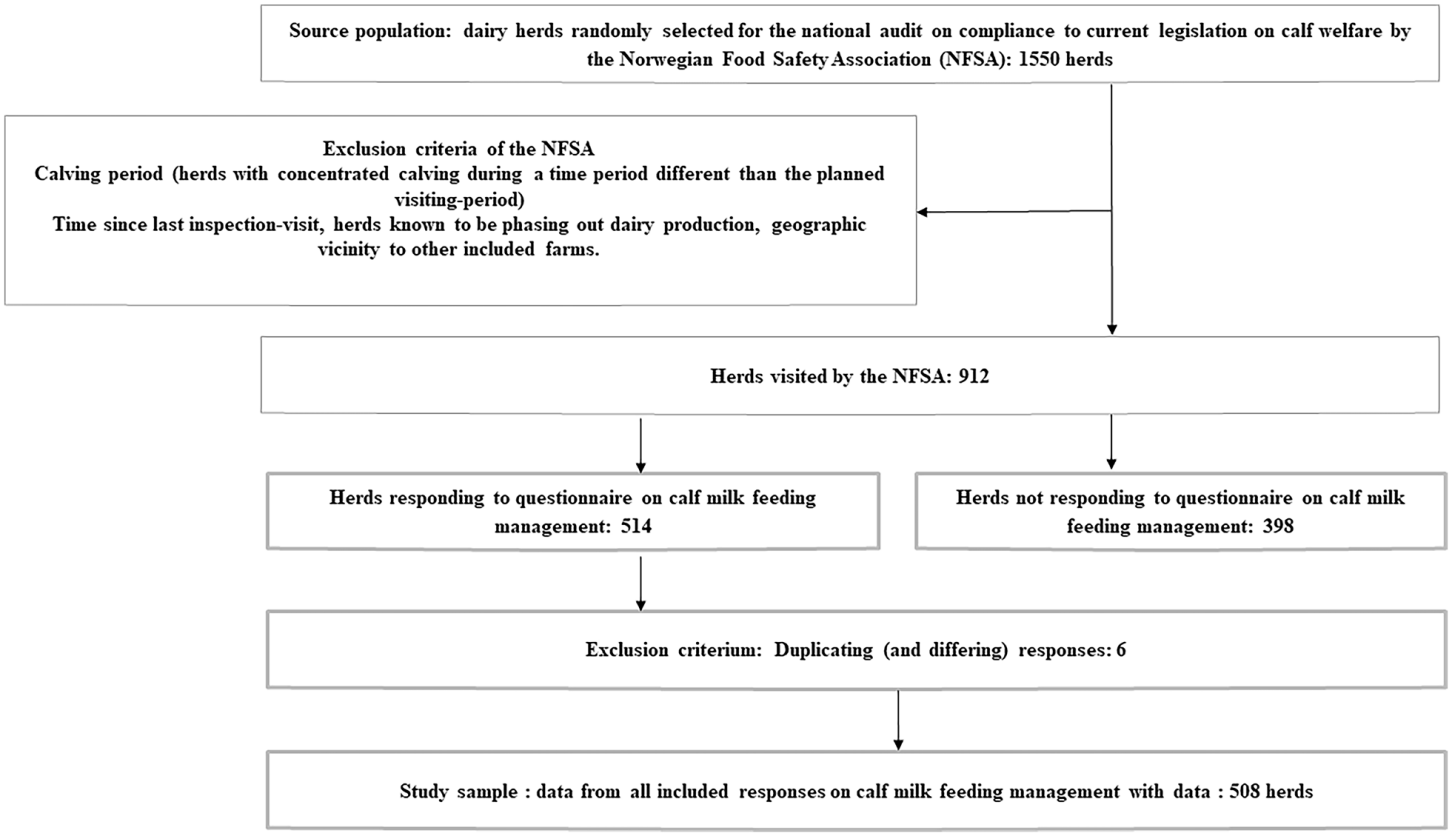
Flowchart of eligible and analyzed herds, and reasons for exclusions

The aim of this study was therefore to describe selected milk-feeding practices and social housing procedures for young (pre-weaning) calves in Norwegian dairy herds. Throughout, “milk" is used to represent both whole milk and milk replacer, unless otherwise specified. From the 9400 Norwegian dairy herds, a random sample of 912 dairy producers received a short questionnaire on housing and milk feeding management in 2016 (Table 1). These producers were visited by veterinary inspectors from the Norwegian Food Safety Authority (NFSA) with the aim to perform a welfare audit as described in [5]. Producers’ input into the questionnaire was voluntary and was obtained either by interview or self-registration. The questionnaires were photographed and returned by mail to the contact person (JFJ), either by the NFSA inspector or directly by the producer.Of the producers receiving the questionnaire, 514 were returned, but 6 questionnaires were excluded (Fig. 1). As a result, this survey contains responses from 508/912 herds (56% response rate). Statistical method is described in Additional file 1.

**Table 1:** Results from questionnaires on herd calf management procedures related to milk feeding (n = 508 herds)

Variable	n	Median	IQR		Min	Max
Daily milk allowance (L/d)	508	7	6	8	2	15
Number of daily milk feedings	501					
Automatic milk feeder	30					
Daily (manual) milk feedings	471	3	2	3	2	7
Missing entries	6					
Housing in single pen (weeks)	504	2	1	3	0	16
Missing entries	4					
Barn type (cows)						
Free-stall	193					
Tie-stall	294					
Free access to water at 3 weeks						
No	82					
Yes	420					
Missing entries	6					
Usage of milk replacer?						
Yes	227					
No	227	2	1	3	0	14
If milk replacer is used, from what age (week)?	227	2	1	3	0	14
Missing entries	10					
	135	Yes				
Has the calf milk allowance been changed during the	345	No				
Missing entries	28					
If yes, what was the former milk allowance?	133	6	5	6	0	9
Missing entries	2

**Fig. 2:**
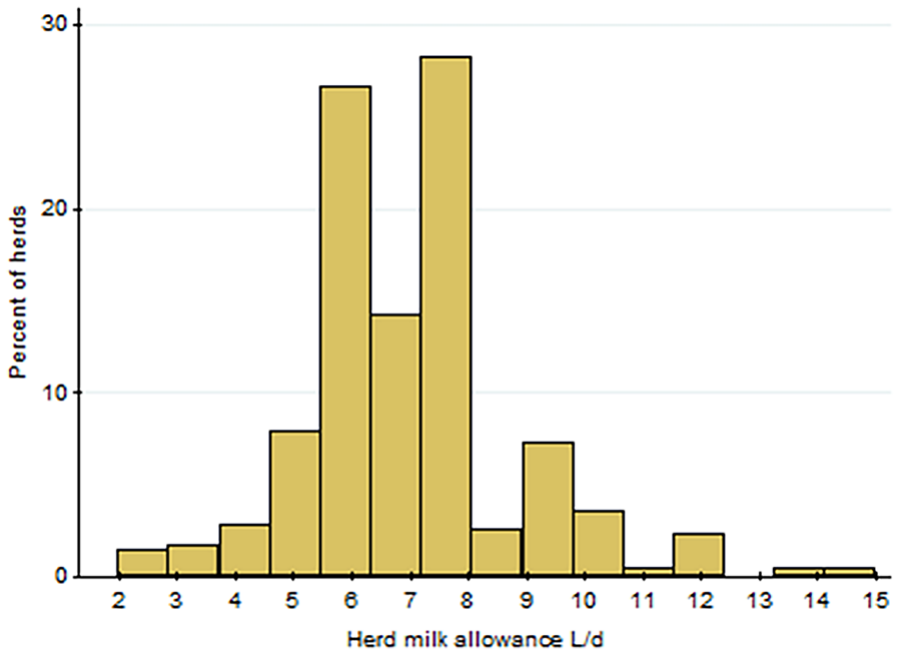
The distribution of dairy herds (n = 508) according to their reported milk allowance to calves aged 3 weeks

Results from this survey showed that median (interquartile range, IQR) milk allowance to 3 week old calves was seven (6–8) L milk/day, ranging from 2 to 15 L/day. Of the study population, 311/508 (61%) herds thus reportedly fed less than 8 L/day to 3 week old calves which is the current industry recommendation in Norway (8 L/d) [6]. Similarly, 76/508 (15%) and 17/508 (3%) herds reported that the calves’ daily milk allowance was less than 6 and 4 L per day, respectively (Fig. 2). Most herds (n = 385, 72%) reported no change in daily milk allowance during the last 4 years. Of the 135/480 (28%) herds that had changed the milk allowance, most had increased the milk allowance (mean difference with respect to current milk allowance was + 1.4 L). Only two herds reported that they had decreased their milk allowance. General descriptions of the surveyed herds can be found in [5].

An automatic milk feeder was used by 30/480 herds (6%) but respondents did not report how the milk feeding frequency was set at their herd. Median number of daily milk feedings in herds feeding milk manually was three. Of these herds, 235/471 (50 %) two times daily, 148/471 (31%) fed three times daily, 64/471 (13%) fed four times daily, 9/471 (2 %) fed five times daily, 12/471 (3%) fed six times daily and 3/471 (1%) fed seven times daily. Of the 226 producers (46%) which reported to use milk replacer, it was used from the calf age of (median) 2 weeks. Of all herds, 82/502 (16%) did not provide their calves with free access to drinking water at the age of 3 weeks. Calves were moved from single pens to group-housing at the (median) age of 2 weeks (IQR 2–3), ranging from age 0–16 weeks. Individual housing beyond 8 weeks was reported by nine herds.

We chose to ask about the milk allowance at the age of 3 weeks. We reasoned that high milk allowances are especially emphasized due to the low capacity to exploit solid feed relative to milk at this age [4]. However, another study found that the critical age span for low milk allowance may be before the age of 3 weeks [6]. Norwegian legislation emphasizes that the feed should promote good health and welfare, and should be adjusted according to the animals’ age, weight, physiological and behavioural needs [7]. A median daily milk allowance of 7 L/day to 3 week old calves is comparable to findings in Canadian farms using manual feeding (7 L/day) [8], slightly higher than Czech (6 L/day) [9] and Californian dairy herds (~ 4L/day) [10] but lower than Canadian farms using automatic feeders (9 L/day) [8]. Our study shows that most dairy herds in the study population provided young calves with less milk than what is currently recommended by the Norwegian dairy industry (8 L/day) [11]. A possible explanation may be that many farmers fear that increased milk meal sizes can cause leakage to the rumen [12]. We found that daily (manual) milk feedings often comply with minimum standards (two feedings) [7]. As practiced by some farmers in the study population, milk feeding frequency may be increased to accommodate higher milk allowances [12]. Several recent studies have highlighted the importance of providing sufficient milk to the young calf [e.g. 4,13,14]. If provided free access to milk from an artificial teat, voluntary milk intakes of young dairy calves are ~10–12 L/day [8, 10]. The results of our survey indicate that many herds still practice to feed restricted milk allowances. Restricted milk allowances are associated with hunger [15]. Behavioural signs of hunger may be many unrewarded visits to the milk feeder or open mouthed vocalizations [15, 16]. Increasing milk allowances allow calves to perform a feeding pattern more consistent with their natural behaviour [17]. Ensuring that dairy calves do not feel hungry is therefore pivotal to prevent poor welfare.

It is now recommended to feed calves higher milk allowances for increased productivity and growth [18–20]. From the perspective of a high first lactational yield, the optimal prepubertal average daily weight gain of Norwegian Red cattle is estimated to 890 g/d [21]. Breed was not recorded in our survey, but 92% of all dairy cows in Norway are of this breed. Optimal pre-weaning growth rates can be expected to be even higher, given that the energy requirement is higher during the first weeks after birth [22] and naturally suckling Norwegian Red calves gain 1.3 kg/d [23]. Knowledge on viable alternatives to restricted feeding regimes is currently expanding [e.g. 12,13,17]. Therefore, feeding more milk should be emphasized to improve not only calf growth, health and welfare, but also future productivity [20, 21].

Most producers that reported to have changed milk allowances during the recent years, had in fact increased the milk allowance. This may indicate changes towards increased allowances. However, the results of our study indicate that there still is considerable room for improvement with regards to the amount of milk fed to calves. Veterinarians are frequently concerned about dairy calf hunger and suboptimal nutrition [24]. New knowledge on dairy calf needs and management strategies that meet these should be disseminated by targeting all relevant stakeholders including dairy producers and veterinarians.

Many producers in our study population did reportedly not make use of milk-replacer. In the herds that did use milk-replacer it was offered to the calves from the age of 2 weeks. This indicates that most calves receive whole milk during the first weeks after birth. We do not have information on the quality of the whole milk (or transition milk) fed to the calves in the current study.

Most, but not all herds reported that 3 week old calves had free access to drinking water. For a subpopulation of the herds surveyed in this study, we recently found an association between the lack of free water access and greater herd calf mortality [5]. Although Norwegian legislation currently renders free access to water compulsory only in case of disease or high temperatures [7], studies show that calves are motivated to drink water [25]. Providing free water access from birth is recognized as an important factor for calf growth and development, potentially by stimulating rumen development [5, 25, 26]. Further, too little fluid (milk or water) will limit the animal’s intake of dry, solid feed such as concentrates [22]. Water access to young dairy calves should thus be emphasized in the future.

Most calves were group-housed before the age of three weeks. According to Norwegian legislation, calves may be housed in single pens up to the age of eight weeks in conventional and one week in organic dairy herds [7]. Some herds (nine) reported grouping calves later than this age. Failure to comply with legislation on calf welfare may result in a NFSA resolution mandating the farmer to resolve the practice as described by the NFSA report from the welfare audit [27]. The finding that calves are grouped at a much lower age indicates an increased awareness with regards to the importance of social housing for calves. Documented favourable effects of social housing are related to cognition, social buffering and social development [3].

An important limitation of the current study was its reliance upon the voluntary response of the target population.

In conclusion, the milk allowances reported in this survey are low compared to industry recommendations which again are low compared to voluntary intakes of young dairy calves. Free access to drinking water is important to calf welfare but was not granted to all dairy calves. Most calves were reportedly group housed at an early age which indicates an improved awareness with regards to the importance of social housing.

## Supplementary Information

**Additional file 1.** Data handling and statistical analysis.

## Data Availability

The datasets used and analysed during the current study are available from the corresponding author on reasonable request.
