# Supplementary material for: A survey of pre-weaning calf management in Norwegian dairy herds
Source: Acta Vet Scand. 2021 May 6;63:20. doi: 10.1186/s13028-021-00587-x (PMC8101324; doi:10.1186/s13028-021-00587-x)
Supplement: Supplementary file 1 — Additional file 1. Data handling and statistical analysis. [file 13028_2021_587_MOESM1_ESM.docx]

**Additional file 1. Data handling and statistical analysis.** Data handling and statistical analyses were performed in Stata (Stata SE/14, Stata Corp., College Station, TX, USA). Some responses were modified for the purpose of analyses. In herds using an automatic milk feeder the number of meals per day was not reported. Therefore, “number of feedings per day” only regards the herds feeding milk manually. In cases where producers reported ranges rather than a fixed quantity (e.g. they fed 7 to 8 L milk/d) the mean of these two data points was used. The distribution of calf daily milk allowance among the herds was assessed using histograms. Centre values and spread for all responses from the questionnaire were calculated using the “summarize, detail” syntax in Stata. Among herds that reportedly had changed their milk allowance during the last three years, we calculated the difference between previous milk allowance and current milk allowance.
